# An evaluation of the public’s Knowledge, Attitudes and Practices (KAP) in Trinidad and Tobago regarding sharks and shark consumption

**DOI:** 10.1371/journal.pone.0234499

**Published:** 2020-06-09

**Authors:** Lauren Ali, Elisabeth Grey, Delezia Singh, Azad Mohammed, Vrijesh Tripathi, Judith Gobin, Indar Ramnarine

**Affiliations:** 1 Department of Life Sciences, Faculty of Sciences and Technology, University of the West Indies, St. Augustine, Trinidad and Tobago; 2 Department of Chemistry, Faculty of Sciences and Technology, University of the West Indies, St. Augustine, Trinidad and Tobago; 3 Department of Mathematics and Statistics, Faculty of Sciences and Technology, University of the West Indies, St. Augustine, Trinidad and Tobago; University of California Santa Cruz, UNITED STATES

## Abstract

There is a global lack of data concerning shark consumption trends, consumer attitudes, and public knowledge regarding sharks. This is the case in Trinidad and Tobago, where shark is a popular culinary delicacy. A Knowledge, Attitudes, and Practices (KAP) survey was conducted in Trinidad and Tobago. Six hundred and seven questionnaires were administered. Univariate and stepwise multivariate logistic regressions were performed to test the association between KAP and demographic categories. The response rate was 93.4% with 567 questionnaires returned (473 from Trinidad and 94 from Tobago). Two hundred and seventeen (38.3%) participants were knowledgeable, 422 (74.4%) displayed attitudes in favour of shark conservation and sustainable use, and 270 (47.6%) displayed practices promoting shark conservation and sustainable use. Island (AOR = 2.81, CI = 1.78, 4.46) and tertiary education (AOR = 2.31, CI = 1.20, 4.46) significantly influenced knowledge level. Gender (AOR = 1.50, CI = 1.02, 2.20) and island (AOR = 0.56, CI = 0.35, 0.90) significantly influenced attitude. Gender (COR = 1.59, CI = 1.14, 2.22) was significantly associated with practices. Over 70% of respondents ate shark, and 54.7% ate shark infrequently enough to avoid risks from heavy metal toxicity. Our results may be useful to develop public awareness and practice improvement initiatives in order to improve KAP regarding shark meat consumption.

## Introduction

According to the IUCN, approximately 16% of shark species are threatened and 20.1% are classified as data deficient [1], and many shark species are particularly vulnerable to overexploitation since they grow and mature slowly [2]. Shark exploitation is driven by international demand for their fins and meat. The economic value and quantity of shark meat on the global market is fairly well understood [3] and may even be used to estimate exploitation levels [4, 5]. However, the cultural norms which drive this exploitation may vary widely, and remain largely unexplored outside of the demand for shark fins. The relationships between shark and consumer may be nutritional, cultural, and economic. Assessment of these relationships are essential for shifting a paradigm of overexploitation to one of sustainability, since understanding and establishing a baseline for consumers’ norms can facilitate opportunities to change these norms [6].

Knowledge, Attitudes, and Practice (KAP) is a framework used to conduct representative studies on specific populations and focuses on investigating what humans know and feel about a topic as well as their associated actions [7]. It has been established that the attitudes of a population can affect its tendencies toward eco-friendly behaviors and policies [8, 9, 10]. KAPs can be used to gauge public knowledge and perception of threatened and exploited species, as well as the public’s current actions and willingness to act in favour of these outcomes, therefore the KAP framework is highly applicable to conservation and management studies.

Several KAP studies concerning fish focus on food safety [11, 12, 13], highlighting links between demographic groups and KAP, investigating consumer preference for certain types of fish, and calculating fish consumption rates. However, they may not address conservation and sustainability concerns. While there are several studies which examine K, A and/or P pertaining directly to sharks and conservation [14, 15, 16], only two studies focused on shark meat consumption and only one of these examined K, A, and P [17, 18].

Trinidad and Tobago is the southern-most country in the Caribbean. Over 30 species of shark are landed by small-scale artisanal and gillnet fisheries [19], however there is very little detail on which species were caught in landing records. The IUCN lists three of the shark species found in Trinidad and Tobago as endangered, nine as vulnerable, and eleven as near threatened. Although shark is mainly bycatch, it has cultural value as a traditional food, and is marketed as such. Shark consumption poses potential health risks since sharks are meso- and apex predators which bioaccumulate heavy metals toxins [20]. While some heavy metals are necessary to the human body in trace amounts, others may adversely affect the liver, central nervous system, bones, other bodily structures, and developing fetuses [21]. Unless excessive, shark consumption on its own may not be sufficient to cause heavy metal poisoning, however, consumption of sharks and other predatory fish may be considered unhealthy due to increased heavy metal intake [22]. The most popular way shark is eaten in Trinidad and Tobago is fried, in a sandwich of fried dough and condiments known as ‘Bake and Shark.’ Two common species in Trinidad and Tobago, *Sphyrna lewini* and *Carcharhinus porosus*, have been shown to contain high levels of the heavy metals mercury, arsenic, cadmium and lead, leading to a recommended consumption rate of less than 272 g, less than once a week [23].

However, no previous studies have examined the frequency and popularity of shark consumption in either island, nor the associated attitudes and behaviors. There has also been no previous assessment of public knowledge or concern regarding endangered shark species, or willingness to adopt practices amenable to sustainable use and conservation (e.g. implement protections for endangered shark species locally). To address this, a KAP approach was used to assess public knowledge of sharks found in Trinidad and Tobago, determine attitudes toward shark consumption, evaluate consumption practices and explore the relationships between these and demographic factors.

## Methods

### Survey instrument

A KAP questionnaire was developed to gather information from citizens of Trinidad and Tobago regarding their knowledge and attitudes regarding sharks and shark consumption patterns, and demographic data (S1 Appendix). A pretest questionnaire was validated with 30 participants from various demographic backgrounds and adjusted as necessary. The questionnaire was divided into 4 parts. The first section consisted of six questions focused on demographics including gender, age, education, place of residence and employment. Gender, age, and education were recorded as stated by the respondent. Place of residence was defined as ‘rural’ if outside a city or ‘urban’ if within a city. Respondents who were classified as ‘employed’ included those who were self-employed or held a paying job. Those who were classified as ‘unemployed’ were those without a paying job. Retirees and housewives did not comprise a large enough segment of the sample population to be classed as separate demographic categories and were therefore included in the category ‘unemployed.’ The following three sections included; (1) seven knowledge-related questions focused on common local shark species, endangered local shark species, anthropogenic and environmental factors influencing local fish and shark populations, and heavy metal contamination in seafood; (2) seven questions regarding attitudes towards consumption of shark meat, consumption and fishing of endangered shark species, species specific labeling of shark meat and consumption of seafood containing heavy metals; and (3) eight questions concerning shark consumption practices (frequency and quantity of shark consumed, decreasing consumption of endangered shark species, consumption of seafood containing heavy metals and willingness to attend a consumer workshop on seafood). To provide context to the shark consumption data, the questionnaire also included six questions regarding frequently purchased fish, venues from which shark is purchased, form in which it is purchased, and shark preparation method. Question types used included multi-option, contingency, five point Likert scale (1-Strongly Agree, 2-Somewhat Agree, 3-Neutral, 4-Somewhat Disagree, 5-Strongly Disagree) and open ended. Redundancy (i.e. asking multiple questions about the same topic in different ways) was used to mitigate social desirability bias in the responses.

A team of interviewers were trained to verbally administer the questionnaire via convenience sampling and assigned to the different regional corporations around Trinidad and Tobago. Twenty-one interviewers conducted surveys in various public places, primarily during weekday afternoons/evenings after and all through the day on weekends. Interviewers approached potential respondents with a brief explanation of the purpose of the survey and its anonymous and voluntary nature. Participants were not allowed to view the questionnaire to avoid them being influenced by the listed potential response options, and interviewer prompting was limited to clarifying the meaning of questions (e.g. defining words) and asking for further explanation from the respondent where necessary. A total of 632 survey attempts were made to counteract the possibility of low participation.

### Sampling design

The target population was defined as citizens of Trinidad and Tobago aged 18 years or older. Minimum sample size was calculated for the country based on a total population of 1328019 (approximately 5% resides in Tobago and approximately 95% in Trinidad) [24] using a 95% confidence interval and a standard error of 0.05), and found to be approximately 385. Stratified sampling was used to produce a minimum sample size per island to ensure representativeness by island, using the formula n_h_ = (N_h_ / N) * n, where h represents a stratum, n_h_ = sample size for h, N_h_ = population size for h, N = total population size, and n = total sample size. This produced a minimum sample size of approximately 18 for Tobago and 367 for Trinidad.

### KAP scores

To evaluate Knowledge, 1 point was assigned to each correct or ‘yes’ response. To calculate Attitude scores 1 point was assigned to responses which were favorable to shark conservation as well as sustainable and safe consumption (referred to in this manuscript as ‘attitudes supporting reduced impact’ of heavy metal consumption and on shark populations). For responses on a Likert scale this meant that one point was assigned to the options ‘strongly agree’ or ‘agree,’ while no points were given for the responses ‘neutral,’ ‘disagree,’ and ‘strongly disagree.’ Shark consumption practices were divided into ‘infrequent’ (less than once per month or not at all) or ‘frequent’ (more than once a month), based on self-reported frequency of shark consumption and purchase of shark. All classifications of shark consumption practice in our manuscript are based on the recommended shark consumption rate for a citizen of Trinidad and Tobago to mitigate exposure to unsafe levels of heavy metals, i.e. less than 272 g, less than once a week [23]. ‘Infrequent’ shark consumption (including no shark consumption) among individuals and households therefore received 1 point while ‘frequent’ shark consumption received no points. Stated willingness to act as an agent of change to promote healthier shark consumption practices and reduce impact on endangered species were also assigned 1 point (this willingness to act, along with infrequent shark consumption, are referred to in this manuscript ‘reduced impact practices’). Scores were summed to produce an individual Total Knowledge Score, Total Attitude Score, and Total Practice Score for each respondent. These summed scores were divided by the number of respondents to calculate mean scores for each section (Knowledge, Attitudes, and Practices) [25]. Percentages were calculated for respondents matching or exceeding the mean scores per section.

Practice data which did not describe frequency and quantity of shark consumption were not included in the calculation of practice score and were considered separately. This was because these data were broadly descriptive of practices linked to shark consumption which cannot be classified in terms of reduced impact/non-reduced impact consumption practices. Therefore, while that data provides valuable context, they were unsuitable for use in the calculation of a mean practice score for shark consumption.

### Data analysis

Data analyses was done using SPSS (version 23). Data was explored using descriptive statistics and normality plots (S2 Appendix). Univariate logistic regressions were performed to examine independent effects of demographic predictors on the dependent variables and all the variables that showed significant influence (p ≤ 0.05) were selected for multivariate step-wise logistic regression analyses [26]. Crude Odds Ratio (COR), Adjusted Odds Ratio (AOR)and 95% Confidence Interval (CI), were also calculated. It should be noted that performing a large number of univariate analyses can increase the possibility of Type I error, however, since this study was being conducted for the first time in the Caribbean, the nature of our analytical approach was exploratory. Further analyses are recommended in the future to confirm the findings of this study.

Data on the frequency of shark consumption were multiplied by the average shark consumption rate per serving of 272g [23] to produce average annual and weekly consumption rates for those who eat shark regularly (at least once per month). Those who ate shark less than once per month were excluded due to minimal health risk.

### Ethics statement

This study was exempt from review under The University of the West Indies Policy and Procedures on Research Ethics, The School for Graduate Studies and Research, February, 2011 which states: “Surveys or Interviews in which researchers’ private data, field notes and published materials are so encoded that there is no likelihood that the identity of the human subjects will be revealed, will normally be exempt from review, unless the nature of the questions could clearly cause distress or even harm. Surveys and interviews of minors (children) are not exempted."

This research was conducted according to the ethical standards of the Life Sciences Department of the University of the West Indies St. Augustine Campus for minimal risk research with human subjects. All participants were informed of the purpose of the study and the nature and duration of the questionnaire, informed that the survey was voluntary, and guaranteed anonymity. As a measure to preserve this anonymity, participants were able to check a box to indicate their consent in lieu of a signature recording their name. No vulnerable populations were targeted in this survey, and all participants were over the age of eighteen i.e. legal adults. The respondents were free to respond/ not respond to any/ some/ all questions and to refuse to continue the survey at any given point of time. In cases where respondents refused to respond to any/some questions, a survey was considered complete if at least 80% of questions were answered, allowing for approximately one unanswered question per survey section in a completed survey instrument.

## Results

### Response rate and socio-demographic traits of participants

Of 632 survey attempts, 607 were successful, producing a response rate of 96%. Surveys that were 80% completed were considered valid, giving a completion rate of 567 (89.7%). Trinidad produced 473 (83.4%) of valid surveys and 94 (16.6%) of valid surveys were from Tobago.

Table 1 shows the demographic composition of respondents. There was no gender bias as males and females were almost equally represented in the sample set. Most respondents were young adults, while the fewest respondents came from the age ranges under 20 years and over 60 years. Over 50% of respondents had a tertiary level education (to which all citizens have free or highly subsidized access), and over 60% were employed. Nearly 60% were from urban areas, and just over 40% were from rural areas.

**Table 1:** Socio-demographic traits used to assess the Knowledge, Attitudes and Practices of respondents regarding sharks and shark consumption in Trinidad and Tobago. The number of respondents per demographic may differ from the total number of persons participating in the surveys (N = 567).

Demographics	Number (%)
**Gender**	
Female	294 (52.4)
Male	267 (47.6)
**Age Range**	
Under 20	21 (3.7)
20–29	183 (32.6)
30–39	116 (20.6)
40–49	100 (17.8)
50–59	78 (13.9)
60 years and over	64 (11.4)
**Education**	
Primary	57 (10.2)
Secondary	201 (36.0)
Tertiary	300 (53.8)
**Employment**	
Employed	347 (62.4)
Not Employed	207 (37.6)
**Area of Residence**	
Urban	326 (58.8)
Rural	228 (41.2)
**Island**	
Trinidad	473 (83.4)
Tobago	94 (16.6)

### Knowledge

The 7 knowledge questions had a total of 37 possible correct responses (i.e. response options which were factual information about sharks in Trinidad and Tobago), producing a mean knowledge score of 8.02 (Standard Deviation 4.74). Two hundred and seventeen (38.3%) participants scored above the mean and therefore were considered more knowledgeable regarding threats to sharks and the status of sharks in Trinidad and Tobago, while 350 (61.7%) scored below the mean.

**Fig 1:**
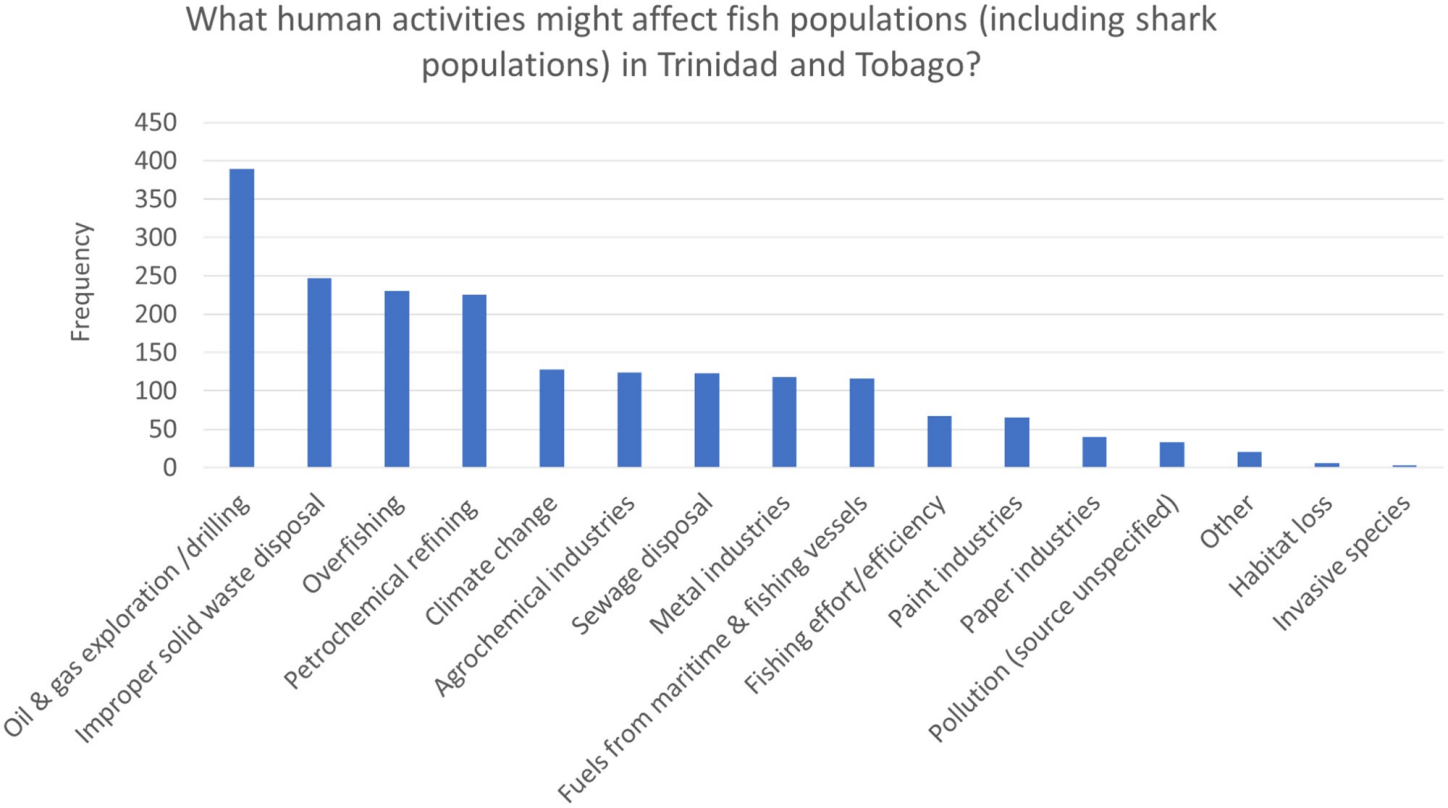
Frequency and percentage of respondents who correctly identified anthropogenic factors which might affect fish/shark populations in Trinidad and Tobago. (See S3 Appendix for Table).

**Fig 2:**
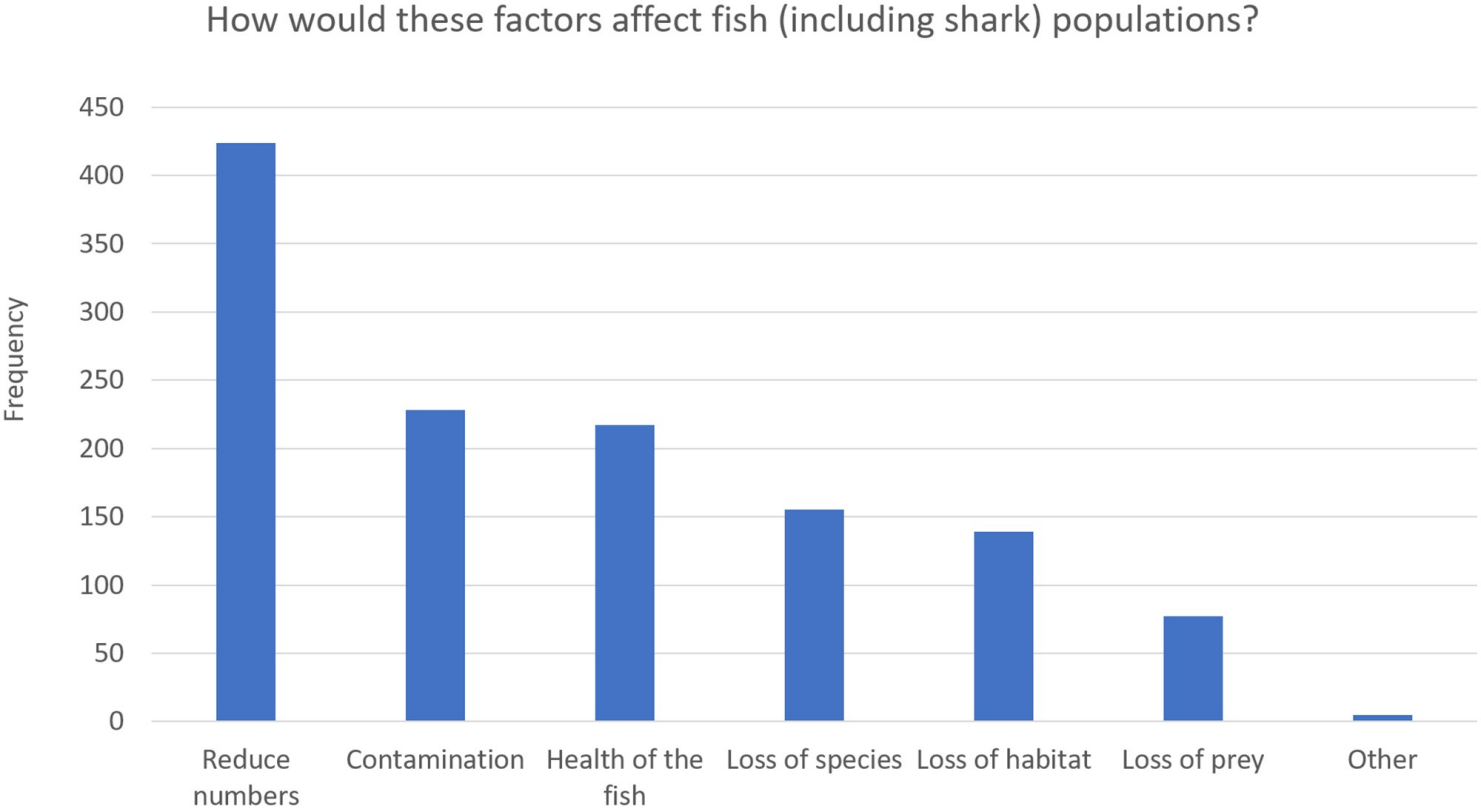
Frequency and percentage of respondents who correctly identified ways in which anthropogenic disruptions might affect fish/shark populations in Trinidad and Tobago. (See S3 Appendix for Table).

**Fig 3:**
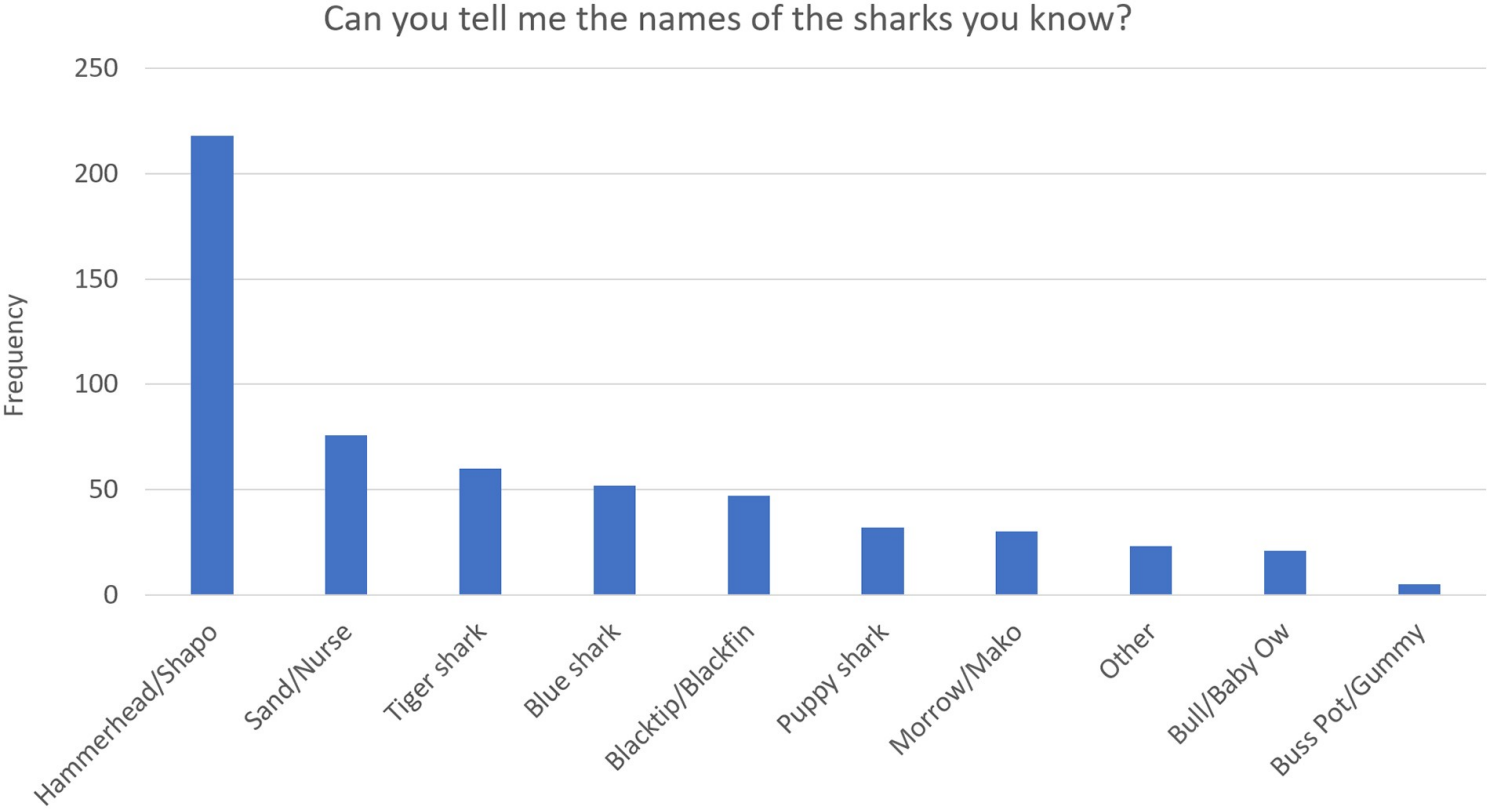
Frequency and percentage of respondents who correctly named types of shark found in Trinidad and Tobago. Names for sharks used in Fig 3 are a combination of common names and local names used in Trinidad and Tobago. Hammerhead/Shapo are Sphyrnidae, Sand/Nurse refers to *Ginglymostoma cirratum*, Tiger shark refers to *Galeocerdo cuvier*, Blue shark refers to *Prionace glauca*, Blacktip/Blackfin refers to *Carcharhinus limbatus* or *Carcharhinus brevipinna*, *‘Puppy shark’* is a catchall term used in Trinidad and Tobago to refer to small Carcharhinid species, Morrow/Mako refers to sharks of the genus *Isurus*, Bull/Baby Ow refers to *Carcharhinus leucas*, Buss Pot/Gummy shark’ are terms used in Trinidad and Tobago which refer to *Mustelus higmani*. (See S3 Appendix for Table).

Table 2 shows the knowledge questions asked in the survey, and along with Figs 1–3 show the frequency distribution of participant’s knowledge responses regarding sharks and environmental factors affecting sharks in Trinidad and Tobago.

**Table 2:** Frequency distribution of participant’s knowledge responses regarding sharks and environmental factors affecting sharks in Trinidad and Tobago (Total N = 567).

Knowledge Questions	Correct/’Yes’ Responses N (%)
Do you think that marine fish (including shark) contain heavy metals?	388 (68.4)
What human activities might affect fish populations (including shark populations) in Trinidad and Tobago? ^a^	530 (93.5)
How would these factors affect fish (including shark) populations? ^b^	516 (91.0)
Do you know how many species of shark are found in local (T&T) waters? ^c^	90 (15.9)
Do you know if there are any endangered shark species in T&T?	122 (21.5)
Do you know of any species/common names of sharks in Trinidad and Tobago?	258 (45.5)
Can you tell me the names of the sharks you know?^d^	257 (45.3)

^a^The values shown in this table represent the number of participants who gave at least one correct response. See Fig 1 for further details.

^b^The values shown in this table represent the number of participants who gave at least one correct response. See Fig 2 for further details.

^c^Since the number of shark species found in Trinidad and Tobago is approximated to be around 30 [17], this question was considered correctly answered if the response was greater than 29 but less than 40.

^d^The values shown in this table represent the number of participants who gave at least one correct response. See Fig 3 for further details.

Table 2 shows that 68.4% of respondents were aware that marine fish contain heavy metals, and only 15.9% of respondents were aware of the approximate number of shark species in Trinidad and Tobago, with 21.5% aware that endangered shark species are present in the country’s waters.

Fig 1 shows the frequency and percentage of respondents who correctly identified anthropogenic factors which might affect fish/shark populations in Trinidad and Tobago. When asked to list anthropogenic factors which might influence populations of marine fish (Fig 1), the most common answer was oil and gas exploration (68.6%), followed by improper waste disposal (43.6%) and overfishing (40.6%).

Fig 2 shows the frequency and percentage of respondents who correctly identified ways in which anthropogenic disruptions might affect fish/shark populations in Trinidad and Tobago. Most respondents stated a decline in numbers (74.8%), followed by contamination of the fish (40.2%) and negative effects on the health of the fish (38.3%).

45.5% claimed they were aware of the names of common sharks found in Trinidad and Tobago (Table 2), but only 24.7% were able to provide these names accurately, and 52.4% of participants were unable to answer the question. Fig 3 shows the frequency and percentage of respondents who correctly named types of shark, specifically those found in Trinidad and Tobago. Species not found in Trinidad and Tobago were included in the category ‘Other.’ The shark most named was the hammerhead (37.4%).

The univariate and multiple logistic regressions showed a significant relationship between knowledge level about threats to sharks/the status of sharks in Trinidad and Tobago and tertiary education, as well as island. The AOR value indicated that those with a tertiary education were 2.31 times as likely to be knowledgeable concerning sharks as those with only a primary education or no education, and that Tobagonians were likely to be 2.81 times as knowledgeable as Trinidadians regarding sharks. Gender, age, employment, and area of residence did not significantly predict knowledge (S8 Appendix).

### Attitudes

**Fig 4:**
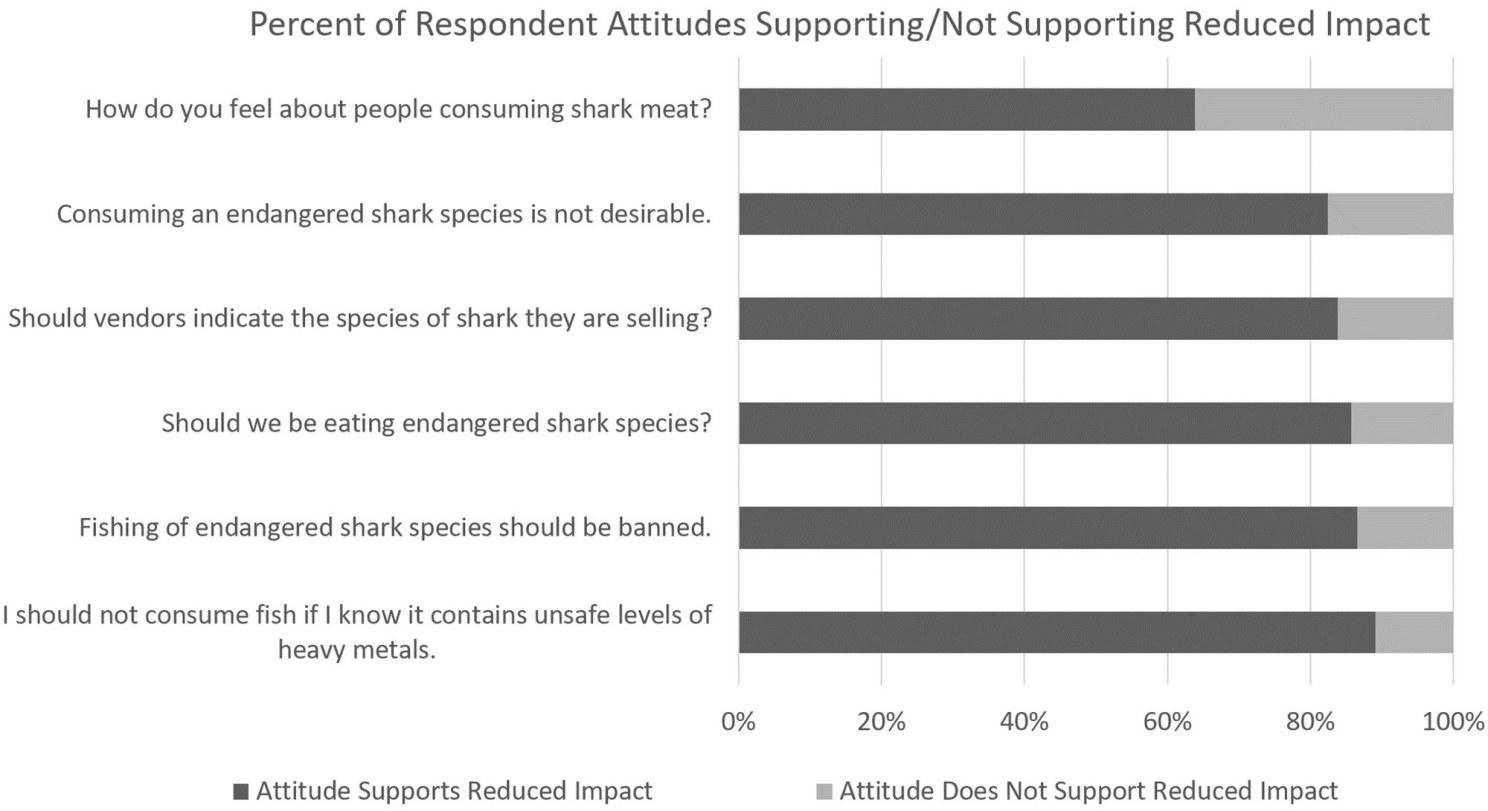
Percent of respondent attitudes supporting/not supporting reduced impact. (See S2 Appendix).

**Fig 5:**
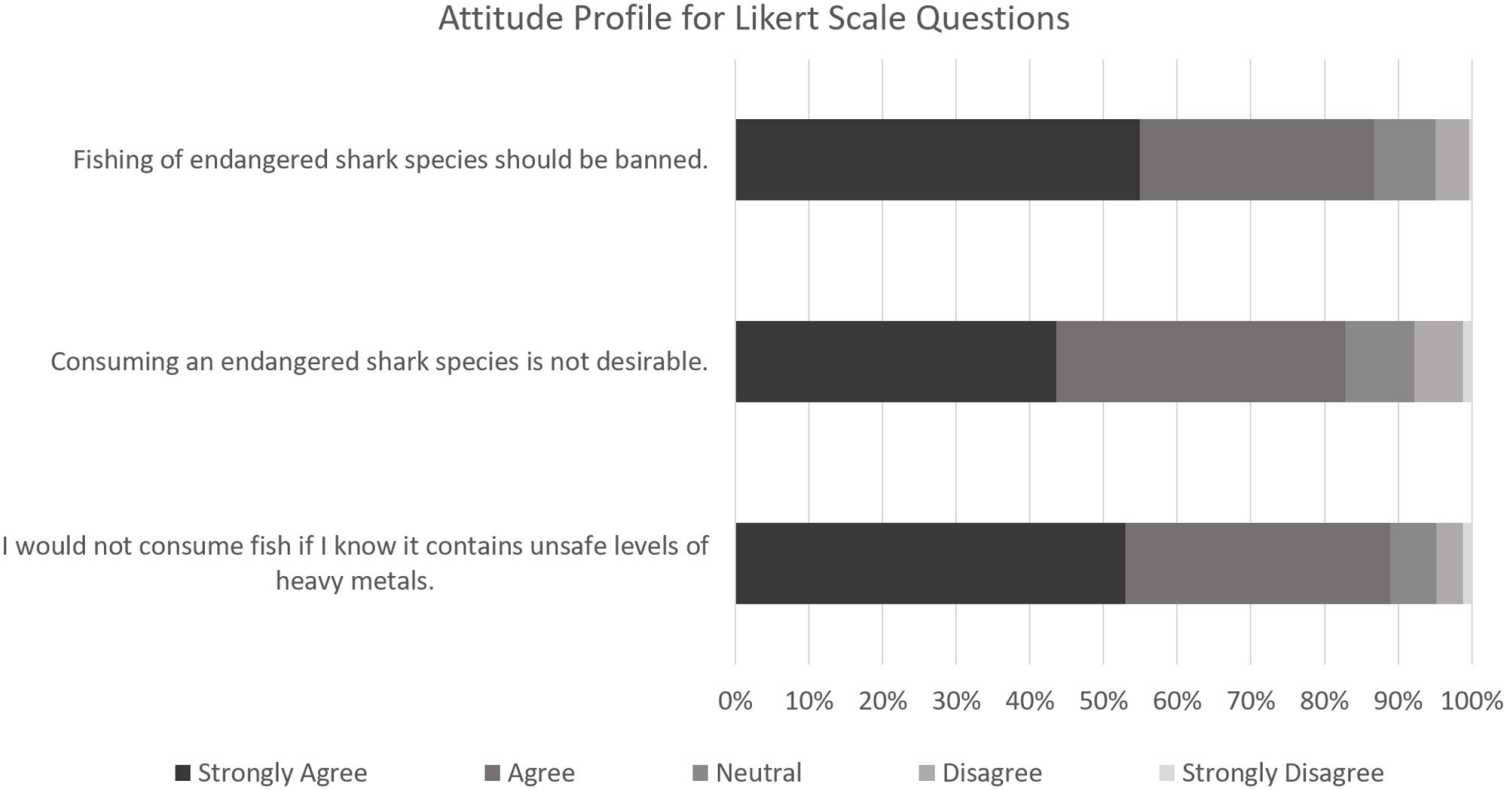
Attitude profiles in response to Likert scale questions. (See S2 Appendix).

Fig 4 shows the attitude questions asked in the survey, and the percent of participant’s attitude responses either in favour of or not in favour of more sustainable shark consumption and heavy metal intake reduction (referred to in Fig 4 as Attitudes Supporting/Not Supporting Reduced Impact). Of six attitude-related questions, three had responses ranked on a Likert scale, shown in Fig 5.

Figs 4 and 5 show that more than 80% of participants received points for almost every question, with respondents mainly not in favour of fishing or eating endangered species, or consuming fish containing heavy metals, and in favour of better labelling practices. The exception was the question concerning the respondents’ support or lack thereof for the consumption of shark meat, with 63.8% of respondents being unsupportive of consumption.

The mean attitude score was 4.91 (SD 1.24). Four hundred and twenty-two (74.4%) of participants had attitudes supporting reduced impacts from shark consumption. The univariate and multiple logistic regressions showed a significant relationship between attitude and gender, and attitude and island. The AOR value indicates that females are 1.54 times more likely than males and Tobagonians were 0.56 times less likely as Trinidadians to have attitudes supporting reduced impacts from shark consumption. Age, education, employment, and area of residence were not found to significantly predict attitude (S9 Appendix).

### Practices

Table 3 shows the practice questions asked in the survey and the number and percentage of participants with reduced impact shark consumption practices. One hundred and sixty (28.2%) respondents did not eat shark, and 117 (20.6%) came from households that did not eat shark. Most of the respondents (85.0%) indicated they would discourage consumption of endangered shark species, 86.1% indicated they would reduce consumption of meat known to be contaminated with heavy metals and 67.4% expressed interest in consumer awareness workshops.

**Table 3:** Number and percentage of participants who had good and poor practices regarding shark consumption.

Variables	Reduced Impact Response	N (%)
Do you eat shark?	No	160 (28.2)
Does anyone in your household eat shark?	No	117 (20.6)
How often do you/does your family eat shark?^a^	Less than once a month	310 (54.7)
How many pounds of shark meat do you/your family usually buy in a single purchase? ^b^	Less than 2 pounds	189 (33.3)
How often do you/your family buy shark? ^c^	Less than once a week	474 (83.6)
If given the chance to attend a community workshop involving consumer awareness of eating fish/shark, would you go?	Yes	382 (67.4)
If you were made aware of heavy metals being present in the fish you bought, what would you do with the fish?	Reduce consumption	488 (86.1)
If you were made aware that some commonly eaten shark species in Trinidad and Tobago were endangered, would you change your consumption habits/ tell others to change their shark consumption habits?	Yes	482 (85.0)

^*a*^Response categorization was as follows: Reduced Impact Practice = does not eat shark, less than once a month; Non-Reduced Impact Practice = once a month, fortnightly, once a week, three times a week, every day (based on recommendations in Mohammed & Mohammed 2017). See S3 Appendix of Table for details.

^*b*^Response options were as follows: Reduced Impact Practice = >1 pound, 1–1.5 pounds; Non-Reduced Impact Practice = 2–2.5 pounds, 3–4 pounds, >4 pounds (based on recommendations in Mohammed & Mohammed 2017 and the average household size for Trinidad and Tobago of 3.24 persons as stated in the 2011 Population and Housing Census Preliminary Count). Shark is sold by the pound in Trinidad and Tobago, so pound increments were selected to correspond as close to practically possible with 0.5 kg increments (rounded up or down). See S3 Appendix of Table for details.

^*c*^Response options were as follows: Reduced Impact Practice = less than once a month, once a month, fortnightly; Non-Reduced Impact Practice = once a week, three times a week, every day (based on recommendations in Mohammed & Mohammed 2017). See S3 Appendix of Table for details.

Table 3 shows that 398 (70.2%) participants consumed shark personally and 424 (74.8%) came from households where shark was eaten. Based on the average household size of 3.24 persons for Trinidad and Tobago [24], and the recommendation to consume less than 272g of shark per person [23], it was found that 265 (46.7%) participants bought relatively large amounts of shark meat (>680 g). Most respondents (54.7%) ate shark once per month or less often, and therefore did not eat shark often enough to cause a public health hazard. The percentage of respondents per island who consumed shark less than once per month was 56.5% in Trinidad and 58.2% in Tobago. Two hundred and twenty-five (39.7%) respondents ate shark at least once per month and among these the average shark consumption rate was 7.6kg annually or 0.635kg per week.

**Fig 6:**
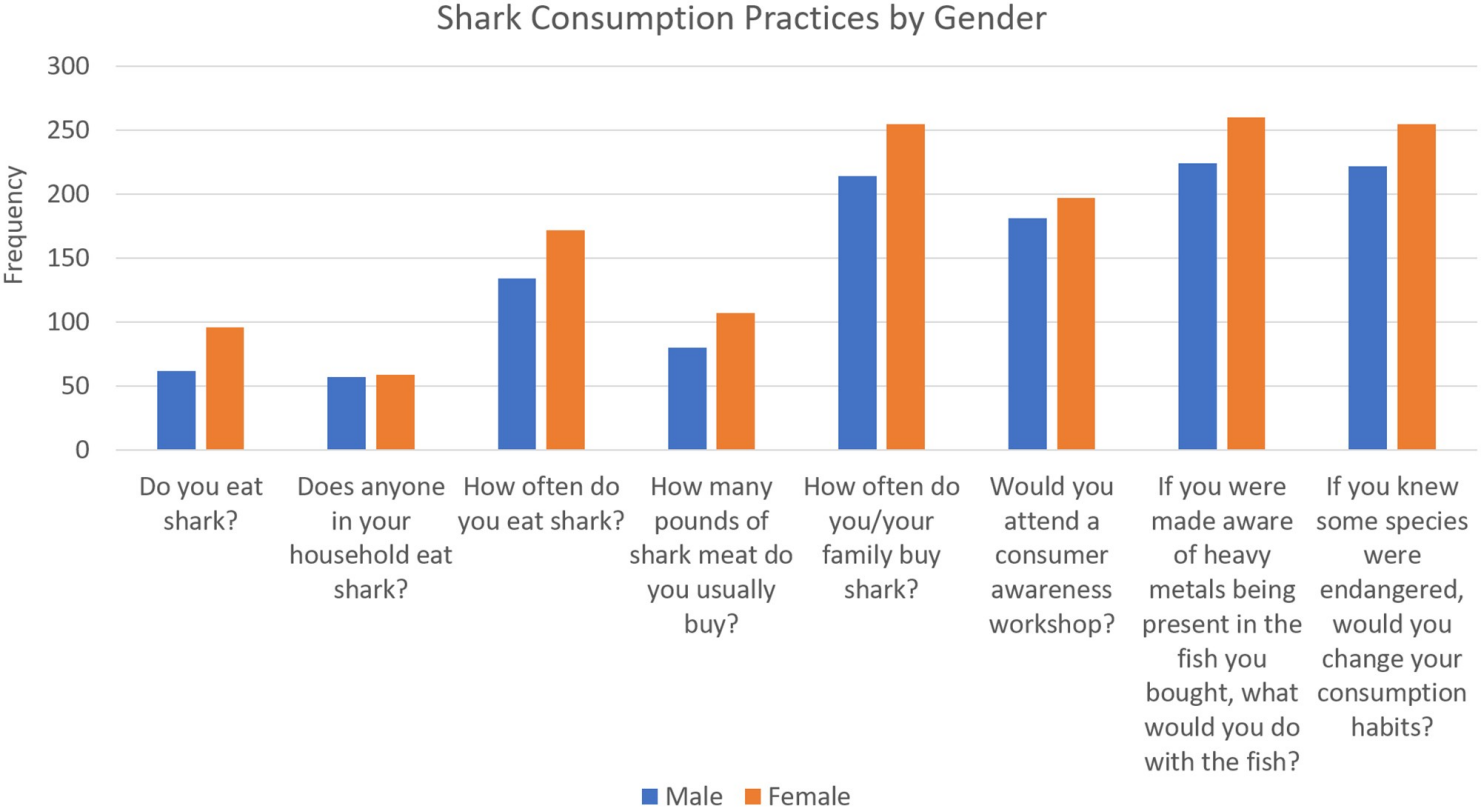
Shark consumption practices by gender.

The mean practice score was 4.58 (SD 1.57), and 270 (47.6%) participants had reduced impact practices. The univariate logistic regression indicated that gender was a significant predictor of practices. Based on the COR, females were 1.59 times more likely to display good practices than males. Age, education, employment, island, and area of residence were not found to significantly predict practices (S10 Appendix). Fig 6 shows shark consumption practices divided by gender.

**Fig 7:**
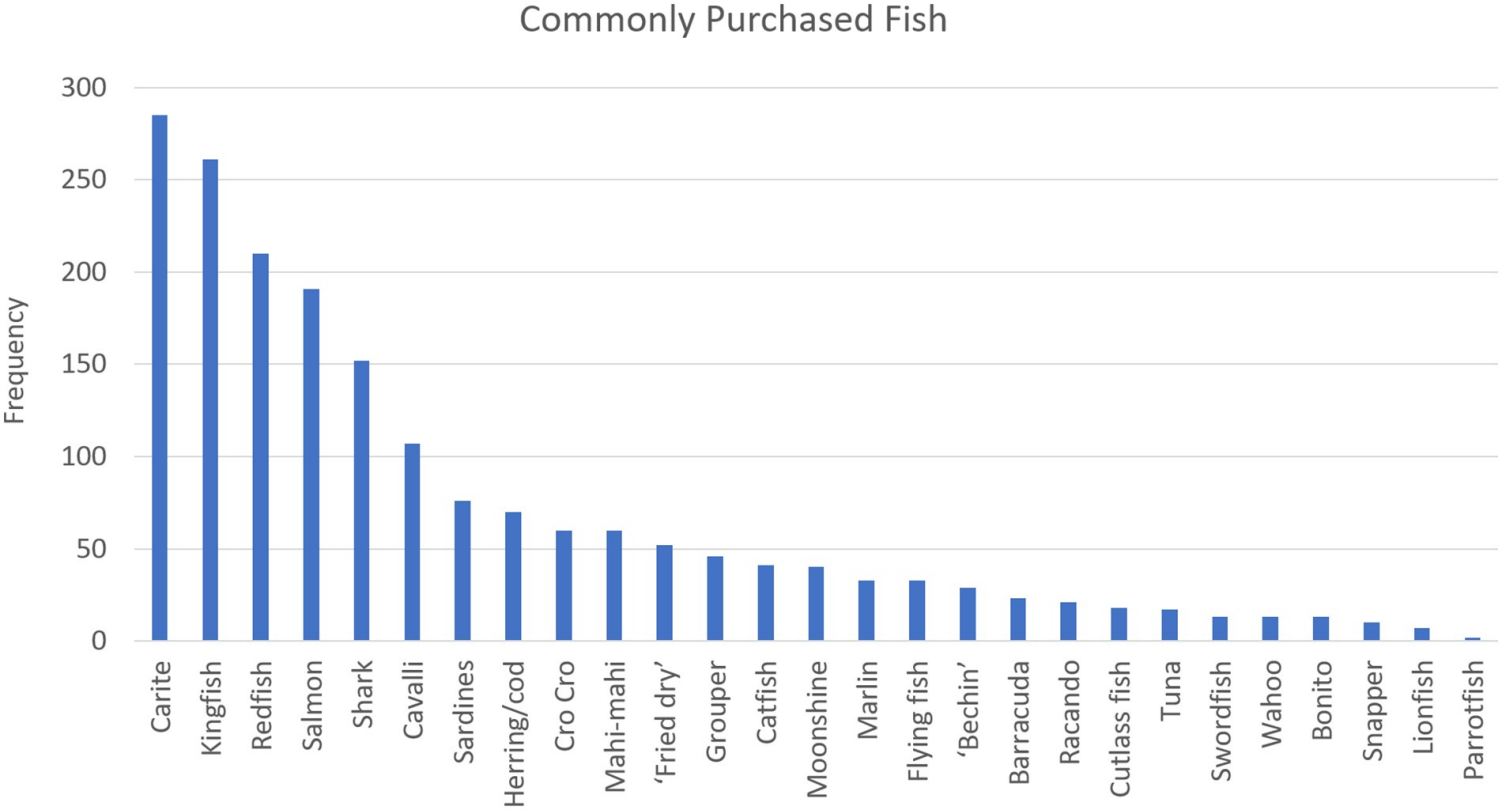
Popularity of different fish among consumers. (See S3 Appendix for Table).

Additional practice data was collected to provide context to shark consumption practices. Fig 7 shows the popularity of different fish among consumers, based on consumer choice. 26.8% of respondents listed shark as a fish that they usually buy, ranking fifth behind carite (50.3%), kingfish (46.0%), redfish (37.0%), and salmon (33.7%).

**Fig 8:**
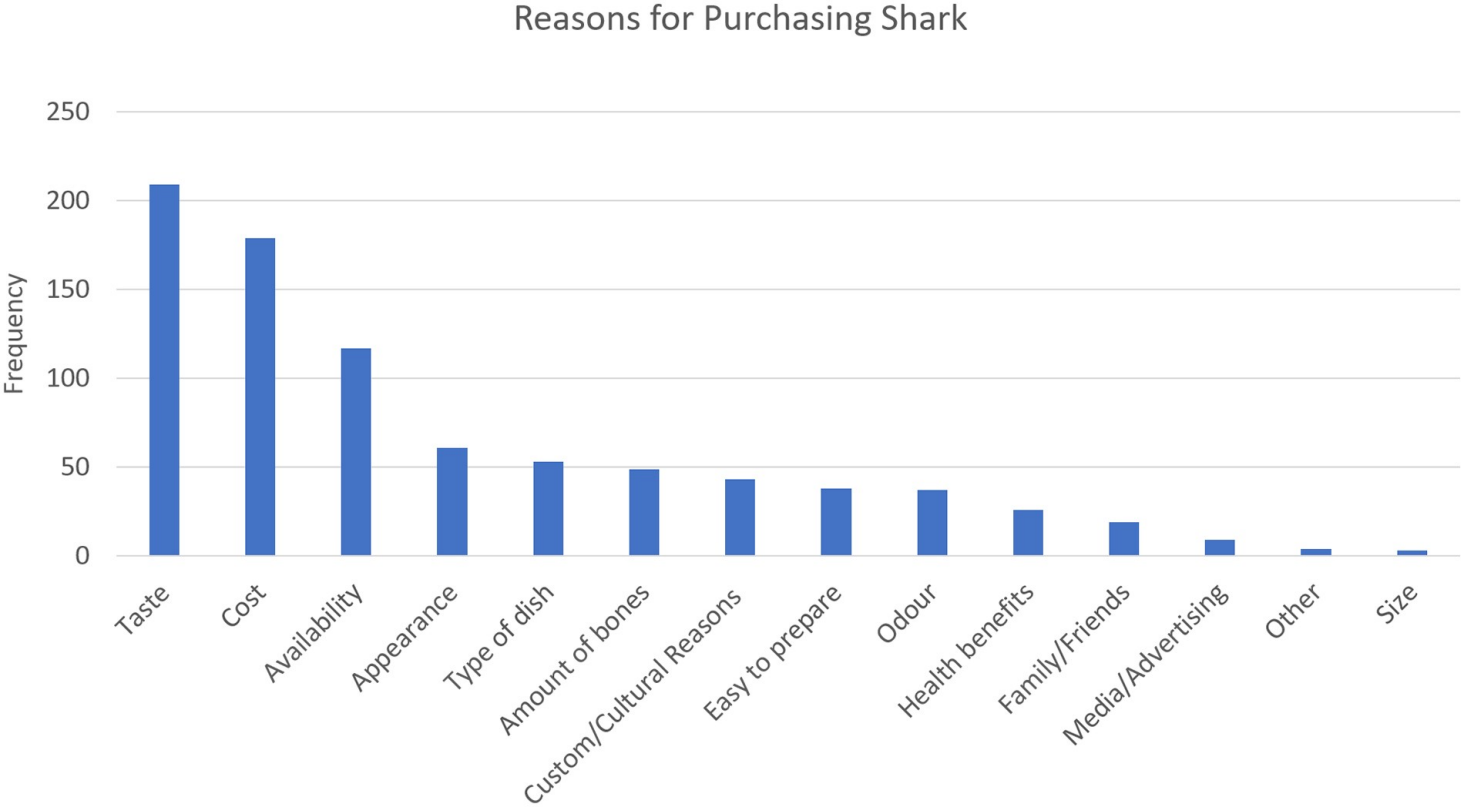
Reasons for purchasing shark. (See S3 Appendix for Table).

Fig 8 shows the reasons respondents gave for why they purchase shark, with taste, cost and availability emerging as the most influential factors.

**Fig 9:**
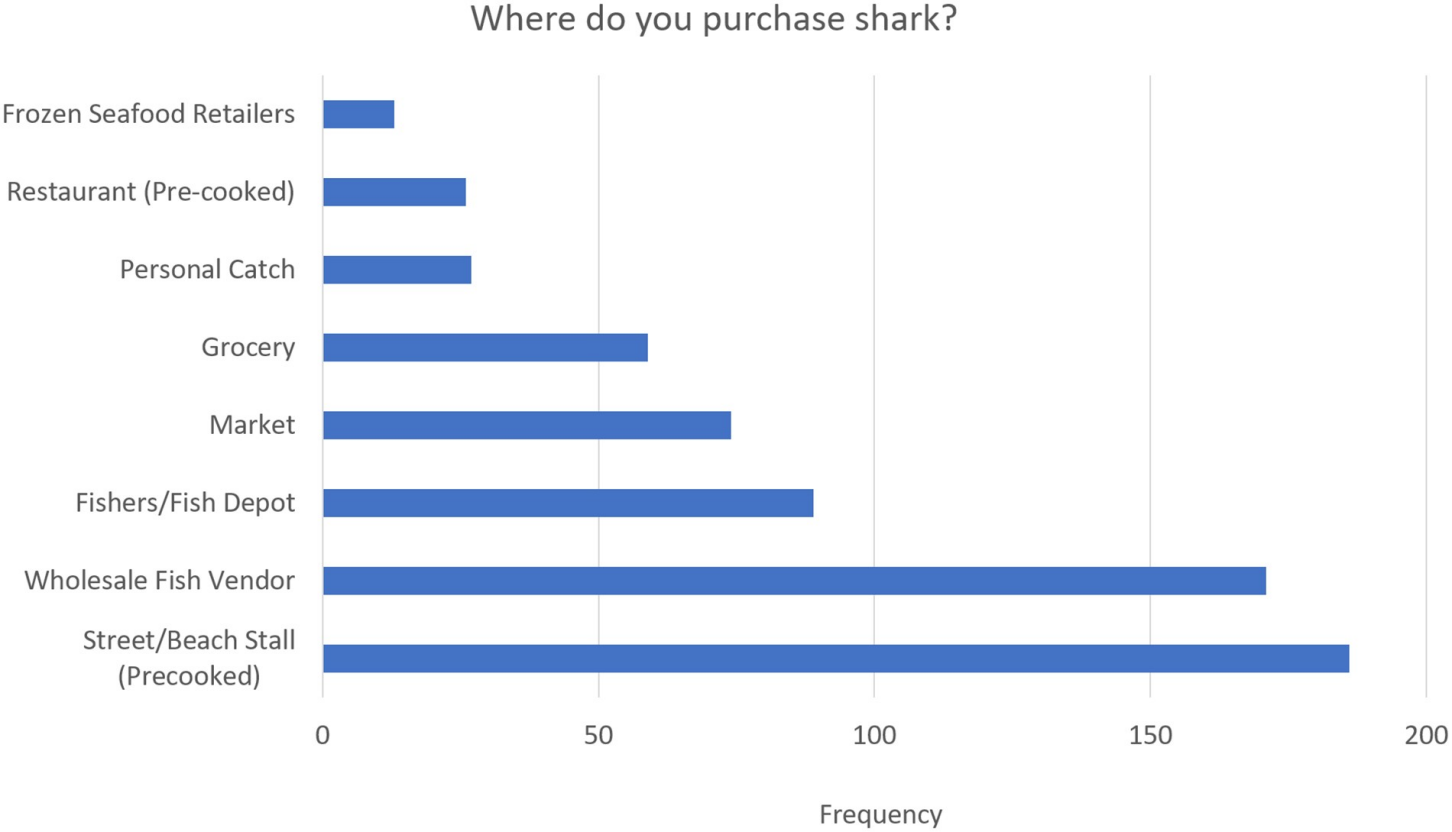
Popularity of different venues for purchasing shark. (See S3 Appendix for Table).

Fig 9 displays the popularity of different venues for purchasing shark, among those who buy it. The majority of shark (32.8%) is purchased cooked from food stalls and the second most popular way to purchase shark is raw, from wholesale fish vendors (30.2%). Third most common are purchases made directly from fishermen (15.7%).

**Fig 10:**
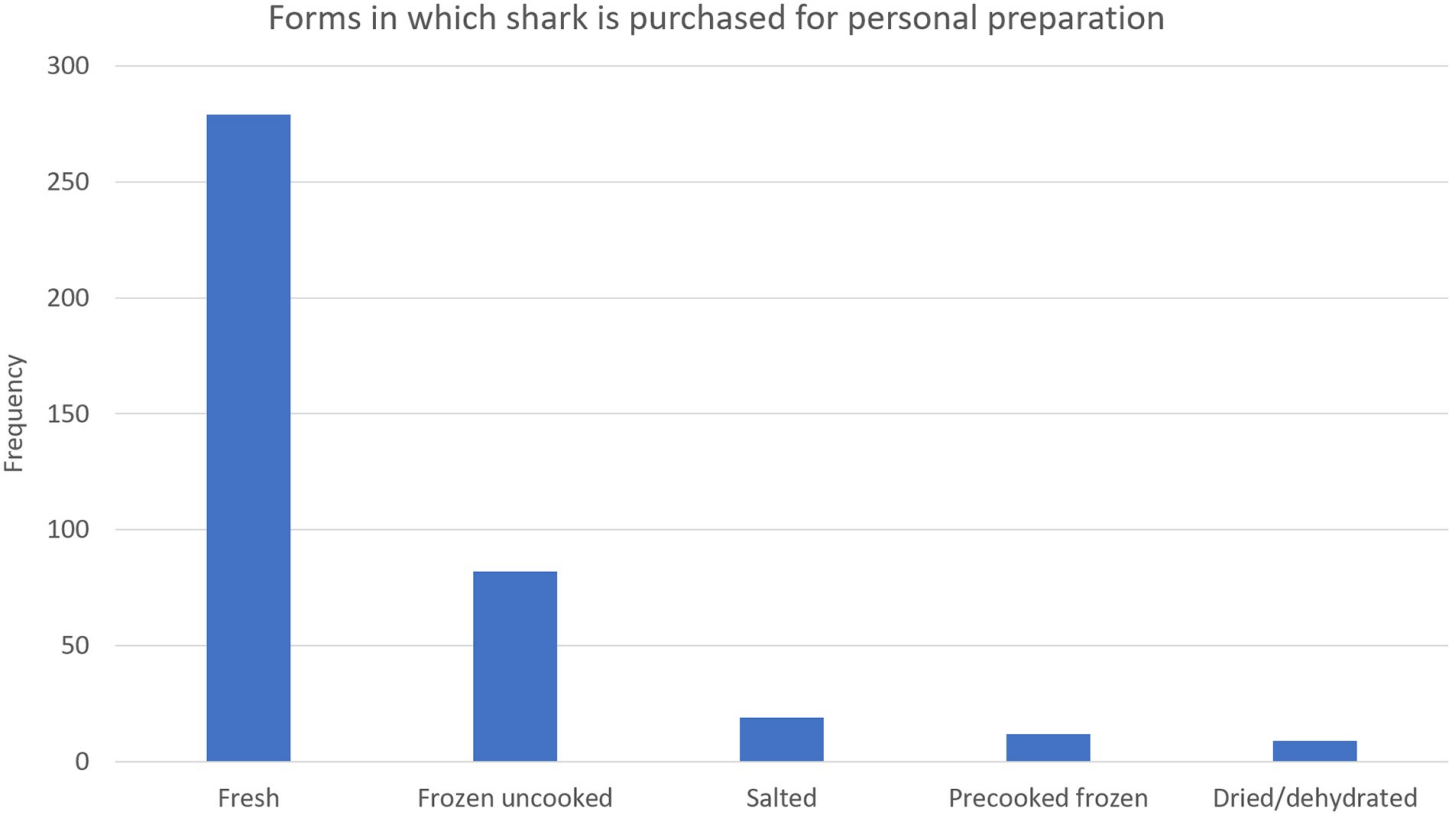
Popularity of various forms in which shark is purchased for personal preparation. (See S3 Appendix for Table).

56.3% of respondents from households that bought shark purchased it to prepare at home. Fig 10 shows the popularity of various forms in which shark is purchased for personal preparation, and shows that fresh shark (84.5%) is most popular among those who prepare it at home.

**Fig 11:**
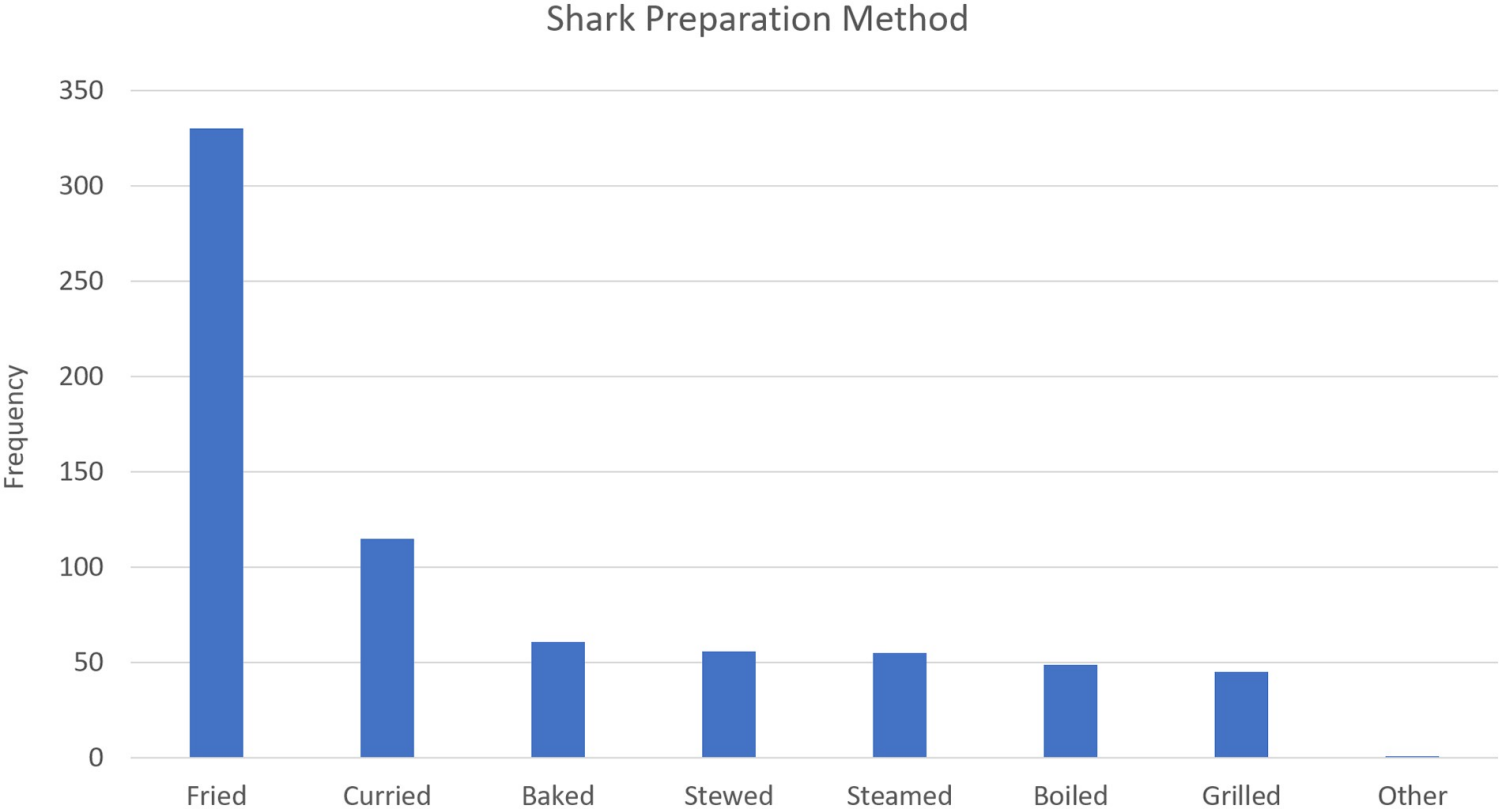
Popularity of methods for cooking shark. (See S3 Appendix for Table).

Fig 11 shows the ways that shark is prepared in the home. Frying is the most popular preparation method, followed by currying, baking, and stewing. It should be noted that frying is the method used to prepare ‘Bake and Shark.’

## Discussion

Overall, fairly few participants were able to name shark species found locally even though most respondents consumed shark. This may be linked to the fact that the greatest percentage of consumers buy cooked shark from food stalls and restaurants (Fig 5), which do not commonly differentiate between species during marketing and sales. This could be improved through stricter labelling practices. Among consumers who buy shark to prepare at home, most buy fresh shark (Fig 7) from fishermen, wholesale vendors, and fish markets (Fig 5). In this context local and common names may be used among salespeople and possibly during interactions with customers, acting as a source of knowledge about local sharks. Participants recognized certain shark species more frequently than others, which may indicate that better recognized species are the most common and therefore the most frequently observed (e.g. in fish markets), or that better recognized species have outstanding physical features (e.g. the hammerhead shark) which make them more memorable, or some combination of both.

Other than personal experience with sharks (at fish markets or otherwise), potential sources of knowledge about sharks include efforts by local conservation groups to highlight shark consumption and conservation, increased information sharing through the internet and social media, television programmes featuring factual or fictionalized depictions of sharks [27], and formal education. Tertiary education was a predictor of good knowledge scores, but did not influence attitudes and practices. Stycos [28] observed that the timing of knowledge acquisition affected practices, where knowledge acquisition at a younger age led to a better practice outcome. Participants may be gaining knowledge about sharks at the tertiary level, after the age at which that knowledge might have the most impact.

Fewer than half of the participants were knowledgeable about sharks in Trinidad and Tobago, but most of the participants expressed better than average attitudes regarding shark conservation as well as sustainable and safe shark consumption. The disconnect between the low percentage of the sample population who were knowledgeable about sharks and the high percentage with impact reducing attitudes may be due to how respondents responded to the questions. Knowledge questions asked respondents to use what they knew to contextualize sharks in Trinidad and Tobago (e.g. conservation status, anthropogenic influences) while Attitude questions presented sharks to the respondents in pre-existing contexts (e.g. endangered species, vessels of heavy metal contaminants). A limitation of this study is that it is possible that respondents may have reacted to these contexts more than to the idea of sharks themselves (e.g. reacting to the idea of endangered species as opposed to endangered *shark* species). Manika [29] found that perceptions of the way in which information is presented influence respondent attitude. In this case the presentation of an unfamiliar topic (sharks) from familiar perspectives (environmental conservation and human health) could be facilitating greater openness to the topic.

Overall, fewer than half of participants exhibited impact reducing practices. It should be noted that the percentage of respondents who consumed shark (70.2%) and the percentage of respondents who claimed they did not support shark consumption (63.8%) were both over 50%. Since all responses are self-reported there is no way to verify the truth quotient of their responses, which may account for this overlap.

### Differences and similarities between Trinidad and Tobago

The significance of island as a predictor of knowledge and attitude may suggest the presence of an underlying cultural difference between Trinidad and Tobago. Trinidadians had significantly better attitudes while Tobagonians had significantly greater knowledge. This may be related to the difference in size and geography between the islands. Tobago (300 km^2^) is smaller than Trinidad (5,131 km²) and contains only about 4% of the country’s population of approximately 1.3 million people [24]. Tobago’s smaller population is concentrated along the coast, since the island contains a central mountain range encompassing a forest reserve. Proximity to the sea was a factor in the selection of sample sites in the study by de la Lama et al. of shark consumption in coastal Peru [18], and it may be that the greater propinquity of Tobago’s population to the ocean (compared to the population of Trinidad) may provide Tobagonians with greater exposure to sharks, facilitating better knowledge scores. Trinidad, on the other hand, is a heavily industrialized island with a large inland population [24]. Trinidadians may therefore experience a greater disconnect from ecological coastal marine resources than Tobagonians, affecting knowledge levels about these topics. These geographical differences may also explain the differences in attitude between the two islands. It is possible that a preponderance of inland, industrially focused livelihood norms may render Trinidadians more amenable to ideas about shark conservation than the coastal Tobagonians, since a smaller proportion of Trinidadians would be associated with marine fishing as a means of livelihood and the associated culture. Further investigation is recommended to assess the possible effects of proximity to, and direct reliance on, coastal and marine resources on knowledge and attitudes toward sharks and shark consumption.

Shark is marketed to tourists in both islands but is generally regarded as a more popular food amongst Trinidadian locals than among Tobagonians. It is therefore interesting to note that there is no significant difference in practices between Trinidad and Tobago, despite the dissimilarities between the two islands in knowledge and attitudes. This may indicate that consumption level is not necessarily linked to the perceived desirability of shark as a food item. For example, it may be that Tobago shark may be consumed more often for subsistence even though it is not very popular, while in Trinidad it is marketed as a delicacy but may be less frequently consumed as a matter of subsistence. However, the stratified, nationwide nature of the present study must be remembered when considering differences between island, since a much smaller number of surveys were conducted in Tobago than in Trinidad. Further investigation is recommended to comparatively examine views on sharks and shark consumption between Tobago and Trinidad.

### Gender and health implications of shark consumption

Females scored better on both attitudes and practices than males. Myrick and Evans [30] reported similar findings in a study examining reactions to videos of sharks, and suggested that in the context of their study the phenomenon might be due to empathy for sharks. While the present study asks some questions (regarding conservation status) which might evoke an empathetic response to sharks, questions also focused on heavy metal contamination as a possible health risk. It should be noted that the minimization of heavy metal intake, including reduced shark consumption, is of particular concern to women due to the risk of foetal heavy metal uptake in addition to the possible effects of heavy metals on the consumer. It could therefore be possible that healthy self-interest might influence Attitudes and Practices in the present study. This is supported by the fact that most respondents, regardless of gender, thought that marine fish contained heavy metals (Table 2) yet gender affected both attitudes and practices. Females may therefore be more open than males to campaigns to reduce shark consumption. Further study of motivations regarding sharks and shark consumption is recommended to ascertain the roles that empathy, self interest and other factors might play in perceptions and actions towards sharks in Trinidad and Tobago. A greater understanding of consumer motivations is necessary to the promotion of safer and more sustainable shark consumption practices.

Although less than 40% of the sample population ate shark regularly, the average individual shark consumption rate among those who ate shark frequently (635g/week) exceeds the recommendation of <272g at the frequency of less often than once per week for reduced risk of heavy metal contamination [23]. This indicates that the popularity of shark promotes unsafe seafood consumption practices. Neupane and Kromash [31] found high levels of mercury in hair samples from Tobago. The population is likely being exposed to heavy metals through a variety of sources, including shark. Considering this, it is recommended that methods be explored to reduce heavy metal concentrations in shark with a view toward reduction in overall heavy metal intake. Frying, the most popular shark preparation method (Fig 7), has been shown to cause a general increase in heavy metal concentrations in fish [32]. However, it was reported to lead to the reduction of lead, iron and chromium in some fish species [33]. It is recommended that heavy metal content in shark tissue be assessed before and after common cooking practices, to determine the possible effects of these cooking practices. The preference for fresh shark for domestic preparation and the high percentage of respondents who indicated they would not want to consume food contaminated with heavy metals suggests potential for a better-informed consumer base to adapt to a cooking method which could reduce heavy metal concentrations.

### The utility of KAP studies for understanding cultural and cross-cultural priorities

The specifics of what constitute good knowledge, attitudes and practices vary based on cultural context [34], which limits the comparability of KAP studies investigating the same topic in different regions. However, regardless of location or cultural context, the KAP framework seeks to examine the relevant components of knowledge, attitudes and practices. Therefore, it could be argued that while the exact components which make up a populations’ KAP may vary widely by location, each KAP is providing a means of assessment of culturally relevant knowledge, attitudes, and practices regarding a specific topic. In combination these cultural snapshots can enable a nuanced vision of the similarities, differences, and variations which define a core issue in varied settings.

Two other studies to date have examined public perception of sharks and the consumption of shark meat (as opposed to fins). Giglio et al. [17] examined knowledge and practices (including consumption) concerning sharks in a small coastal area of east Brazil, and de la Lama et al. conducted a KAP study on coastal populations in Peru [18] around the same time period as the study in Trinidad and Tobago. The conditions under which de la Lama et al. [18] conducted their study and the analyses used by Giglio et al. [17] bear some relevant similarities to those of the present study, however, the conditions and methodologies were not identical. While only some questions could be considered similar enough for direct comparison between studies, they all investigate relevant cultural factors surrounding sharks and shark consumption and produce some similar results.

Our study and de la Lama et al. [18] both found that fewer than half of the participants could name shark species present in their respective countries, and certain species were better recognized than others. Giglio et al. [17] found that slightly less than half of respondents could name local endangered species, while our study found that just over one fifth of respondents were aware of local endangered species. Hammerhead sharks were among the best recognized types of shark in both Giglio et al. [17] and our study. These similarities between studies point to a general lack of knowledge about shark species, and a disconnect from local marine environments and marine food sources despite the proximity of the various target populations to the sea.

Approximately 70% of the sample populations in both Trinidad and Tobago and Peru were involved in shark consumption, and in east Brazil 94% of respondents ate shark monthly. Shark consumption was prevalent in all three locations, providing insight into fishing pressure affecting shark populations along the South American coast. Shark appears to be a prevalent food source along the South American Coast despite the widespread lack of knowledge of shark species. It is interesting to note that in both Peru and Trinidad and Tobago shark consumption is common, but it does not seem to be a staple food. This may be due to factors such as availability, cost, and culture. For example, in Trinidad and Tobago shark is often marketed in association with recreational activities as opposed to as a daily meal, and in Peru it may be sold under the trade name ‘tollo,’ which consumers may be unaware is in fact shark.

Some differences in the results of the studies were as follows: in Trinidad and Tobago females had better attitudes than males, while the reverse was observed in Peru; de la Lama et al. [18] found that older participants were more likely to know the names of sharks found in Peru while our study found that age had no significant impact on broad knowledge about sharks; Education levels impacted knowledge but not attitudes in Trinidad and Tobago, while education levels did impact attitude in Peru [18]. Disparities between the results of the studies may therefore be related to cultural differences between sample populations a well as differences in methodology.

More importantly, the three studies produced an overall assessment of the knowledge, attitudes, and practices most relevant to their context and allow a broad comparison of priorities between disparate regions despite their differences. An expanded body of literature would create the opportunity for assessment of cross-cultural vs. location-specific priorities, a notion that can be applied broadly to the use of KAPs in conservation and sustainability studies.

### Recommendations for improving Knowledge, Attitudes, and Practices to encourage sustainable and safe shark consumption

Knowledge, Attitudes, and Practices have a complex non-linear relationship [35, 36, 37]. This should be kept in mind when devising interventions based on KAP data since K, A, and P should be considered individually despite overlaps. The broad target population [38, 39], in this case ‘shark consumers,’ should also be segmented into target groups, and we have suggested some based on our results. To be impactful, all the recommended interventions should first establish a baseline to which the intervention outcomes could be compared, and could use tools such as the before–after–control–impact (BACI) framework, in combination with matching techniques to reduce the effects of bias, gauge outcomes [6].

Several interventions are recommended to improve K, A, and P. The first is a follow-up survey to understand the sources of differences in attitude score between males and females and between Tobago and Trinidad, and to explore the reasons for attitudes towards sharks and shark conservation among the general population. Using methods such as word association [18] greater insight can be gained into the differences in perception of sharks as both animal and food source. It would also be useful to investigate the type, frequency, and effects of interactions between respondents and sharks, since nature experiences generally have a positive effect on attitudes and behaviour [40]. Possible reasons for gender differences in attitudes towards sharks may include fear and incorrect perceptions of abundance, as found by Acuña-Marrero et al. [16]. A greater understanding of what motivates attitudes could then be used to design interventions to increase knowledge as well as to affect behaviour change.

Perceived knowledge has been shown to interact with attitudes [41] and influences actions [42], therefore an educational campaign is recommended to reduce ignorance among shark consumers and provide them with the ability to make informed shark consumption choices. Such a campaign should cover the species of sharks present in Trinidad and Tobago, with special emphasis on their conservation status and on endangered species, as well as the danger of heavy metal contamination and sustainable alternatives to shark consumption. Although age did not affect knowledge, however, children could be considered a priority target group [28]. Outreach to primary schools is recommended to reach the greatest number of children since nearly all respondents had experienced primary education. While a portion of this outreach could involve traditional classroom learning [15], greater emphasis should be placed on increased opportunities for children to engage in nature based experiential learning, which can facilitate increased connectedness to nature and influence environmental behaviors [43]. Women are a target group of particular public health interest due to the risks of foetal heavy metal toxicity. Increased knowledge may reinforce the prevalently impact reducing attitudes and practice displayed by women in the sample population [41, 44]. An education campaign could therefore utilize local media such as radio and television programs targeted at women, as well as reaching out to women’s groups and female health care facilities. Two other target groups are Trinidad and Tobago respectively, since there was a significant difference between the two locations. Cultural differences between the islands should be considered. For example, Tobago is traditionally the more tourist oriented of the islands, therefore an education campaign in Tobago might emphasize the economic value of sharks as a source of eco-tourism (e.g. opportunities to dive with sharks). In Trinidad, which suffers from greater marine industrial pollution, it might be more effective to emphasize heavy metal contamination. Although the present study suggests explanations for the causes of the significantly higher Knowledge scores in Tobago as opposed to Trinidad, further investigation is recommended in order to devise effective education strategies.

Strategies which may influence decision making behavior include rewards, penalties, manipulation of choice architecture (i.e. the layout of options presented to consumers, aimed at impacting their decisions), and social marketing. Provision of benefits as an incentive for pro-conservation behavior has not been shown to be maximally effective, with greater efficacy based on the nature of the reward and occurring when individuals benefit directly instead of as a community [45]. Instituting penalties for irresponsible behavior may change the frequency of that behavior, but does not necessarily build a sense of social responsibility around sustainable resource use [46]. Adjusting the choice architecture around shark purchase is a more effective approach for affecting consumer interactions [47], but faces an obstacle in implementation when applied to reducing shark purchase since it would require the those selling shark to discourage purchase of their own product. To both improve consumer practices and affect a market driven change in shark consumption, a social marketing approach might be most effective. To do this it is necessary to understand the context specific nutritional, cultural, or economic value which sharks and shark consumption provide to each of the target groups (in this case the different levels of gender and island). Alternative behaviours can then be promoted which provide replacement value for less healthy or sustainable practices. Promotion should follow a Theory of Change, be executed through the most effective media for the target group, and may include the creation of catchy slogans, appealing characters or arresting visuals [48].

## Conclusion

Understanding the local context of sharks is essential to planning effective sustainable management and conservation initiatives [49, 50, 51]. In Trinidad and Tobago, shark consumption is prevalent but not frequent, and consumers generally knew very little about sharks even though they had good attitudes towards sustainable shark use and the public health implications of shark consumption. Tobagonians knew more about sharks, but Trinidadians had attitudes more open to safe and sustainable shark consumption. Tertiary education was a good predictor of knowledge about sharks but failed to predict attitudes or practices. Both the attitudes and practices of women were more inclined towards safe and sustainable shark consumption than those of men. This study identified demographics of concern with regard to Knowledge (tertiary education and island), Attitudes (gender and island) and Practices (gender) surrounding sharks and shark consumption which can be used to establish target groups for intervention efforts such as education initiatives, research into the reasons for attitudes towards sharks and shark consumption, and social marketing campaigns to promote safer and more sustainable shark consumption and shark conservation. It also represents a significant contribution to the small body of literature surrounding KAP as it relates to the consumption of shark meat, facilitating a degree of cross-cultural comparison of the practice.

## Supporting information

S1 Appendix KAP questionnaire. (PDF)

S2 Appendix Data exploration. (XLSX)

S3 Appendix Tables used to create bar charts. (XLSX)

S4 Appendix Knowledge data. (SAV)

S5 Appendix Attitude data. (SAV)

S6 Appendix Practice data 1. (SAV)

S7 Appendix Practice data 2. (XLSX)

S8 Appendix Knowledge regression results. (PDF)

S9 Appendix Attitude regression results. (PDF)

S10 Appendix Practices regression results. (PDF)
