# Supplementary material for: An evaluation of the public’s Knowledge, Attitudes and Practices (KAP) in Trinidad and Tobago regarding sharks and shark consumption
Source: PLoS One. 2020 Jun 9;15(6):e0234499. doi: 10.1371/journal.pone.0234499 (PMC7282724; doi:10.1371/journal.pone.0234499)
Supplement: S1 Appendix — (PDF) [file pone.0234499.s001.pdf]

THE UNIVERSITY OF THE WEST INDIES
ST. AUGUSTINE, TRINIDAD AND TOBAGO, WEST INDIES
FACULTY OF SCIENCE AND TECHNOLOGY
DEPARTMENT OF LIFE SCIENCES
Telephone: (868) 662-2002, Ext. 83095/83789/83111/82045 Fax: (868) 663-5241

KNOWLEDGE, ATTITUDES AND PRACTICES ON CONSUMPTION PATTERNS OF COMMERCIAL FISH AND SHARK MEAT IN TRINIDAD AND TOBAGO 2016.

Authorization has been granted by the Department of Life Sciences,
Faculty of Science and Technology,
The University of West Indies
St. Augustine

Information collected from this questionnaire will be used ***for research purposes of only.*** *All information acquired will be treated with confidentiality and managed under supervised protocols.*

Academic Supervisors:

-Dr. Azad Mohammed (The Faculty of Science and Technology, The UWI)
-Dr. Terry Mohammed (The Faculty of Science and Technology, The UWI)
-Dr. Judith Gobin (The Faculty of Science and Technology, The UWI)

**COMPLETION OF THIS SECTION SHOULD BE DONE <u>BEFORE</u> INITIATING THE INTERVIEW WITH THE INTERVIEWEE.**
**Interviewer and questionnaire no.:**
**Location of survey:**
**Date:**
Participatory Response (please tick based on whether or not the interviewee agrees with the following statement, and record a reason given by respondent if refusal was selected).<br><br>"I confirm that the purpose of the research, the study procedures, and any possible risks or discomforts, as well as the possible benefits that I may receive by taking part in the study, have been explained to me. Alternatives to my taking part have also been discussed and all my questions have been answered. I further confirm that I have been provided with the telephone number(s) of the Principal Investigator for contact in the case of an emergency. I confirm that I am willing to take part in this study."<br><br>**Responded** ☐<br>**Refused** ☐
**DEMOGRAPHIC INFORMATION OF INTERVIEWEE**

**Interview category/question**	**Interviewee's responses**	**Guidelines for interviewers**
Gender	Male ☐<br>Female ☐	
What age bracket do you belong to?	Under 20 ☐<br>20-29 ☐<br>30-39 ☐<br>40-49 ☐<br>50-59 ☐<br>60 and over ☐	
What is you level of education at present?	Tertiary ☐<br>Secondary ☐<br>Primary ☐<br>None ☐	
Where do you currently live?	Urban ☐<br>Rural ☐<br>(specify)____________	Interviewer can write down the name of the location if given.

Are you currently employed?	Yes ☐<br>No ☐<br>Other (specify) ☐<br>____________________	
If 'Yes' for the above, state your occupation	____________________	
**A. KNOWLEDGE ON CONSUMPTION PATTERNS, BENEFITS AND RISKS**		
Do you think that marine fish contain heavy metals?	Yes ☐<br>Not sure ☐<br>No ☐	**Interviewers can describe what a heavy metal is** (WHO 2011) -<br>1) a metallic element<br>2) persistent in the environment for extended periods of time.<br>Example – lead
What human activities might affect fish populations (including shark populations) in Trinidad and Tobago?	Petrochemical refining operations ☐<br>Oil & gas exploration /drilling ☐<br>Metal industries ☐<br>Improper solid waste disposal (batteries, fluorescent bulbs,brake pads) ☐<br>Paper industries ☐<br>Paint industries ☐<br>Climate change ☐	Agrochemical industries (fertilizers) ☐<br>Fuels from maritime & fishing vessels ☐<br>Overfishing ☐<br>Fishing effort/efficiency ☐<br>Sewage disposal ☐<br>Other (specify) ☐<br>________________<br>Not Sure ☐<br>Not being affected ☐
How would these factors affect fish (including shark) populations?	Reduce numbers ☐<br>Contamination ☐<br>Loss of species ☐<br>**Health of the fish** ☐<br>Loss of prey for larger fish ☐<br>Loss of habitat ☐<br>Not sure ☐<br>Other (specify) ☐<br>____________________	**'Health of the fish'** - this can include effects related to reproduction and /or spawning also. Details can be recorded here as given by the participant.
How many species of shark are found in local (T&T) waters?	0-9 ☐<br>10-19 ☐<br>20-29 ☐<br>30-39 ☐<br>Not sure ☐	

Do you know if there are any endangered shark species in Trinidad and Tobago?	Yes<br>No<br>Not sure	**Endangered species-** "considered to be facing a very high risk of extinction in the wild" (IUCN Red List ver.3.1 2001).
Do you know of any species/common names of sharks in Trinidad and Tobago?<br><br>**If 'Yes' to previous question** Can you tell me the names of the sharks you know?	Yes<br>No<br>Not sure<br><br>Puppy<br>Hammerhead/Shapo<br>Blue shark<br>Bull/Baby Ow<br>Blacktip/Blackfin<br>Mako/Morrow	Sand/Nurse<br>Buss Pot/Gummy<br>Tiger<br>Not sure<br>Other (specify)<br>______________
**B. ATTITUDES ON CONSUMING SHARK MEAT AND RISKS ASSOCIATED WITH CONTAMINATED COMMERICAL FISH CONSUMPTION.**		
How do you feel about people consuming shark meat?	I support eating it<br>I do not support this<br>Not sure	
Should vendors indicate the species of shark they are selling?	Yes<br>No<br>Not sure	
Should we be eating endangered shark species?	Yes<br>No<br>Not sure	
**Likert scale questions with levels of agreement. Interviewers need to indicate to interviewees that a series of statements need only read the bold statement once the interviewee is aware of the levels of agreement from which they can choose.**		
**I should not consume fish if I know it contains unsafe levels of heavy metals.**<br><br>Do you agree or disagree or are you neutral about this statement?	Strongly agree<br>Somewhat agree<br>Neutral<br>Somewhat disagree<br>Strongly disagree	

**Consuming an endangered shark species is not desirable.** Do you agree or disagree or are you neutral about this statement?	Strongly agree ☐<br>Somewhat agree ☐<br>Neutral ☐<br>Somewhat disagree ☐<br>Strongly disagree ☐	
**Fishing of endangered shark species should be banned.** Do you agree or disagree or are you neutral about this statement?	Strongly agree ☐<br>Somewhat agree ☐<br>Neutral ☐<br>Somewhat disagree ☐<br>Strongly disagree ☐	
**BEHAVIOUR ASSSOCIATED WITH SHARK AND COMMERCIAL FISH PURCHASE, PREPARATION AND CONSUMPTION**.		
What marine fish do you normally buy?	Carite ☐<br>Kingfish ☐<br>Redfish ☐<br>Grouper ☐<br>Shark ☐<br>Salmon ☐<br>Cavalli ☐<br>Lionfish ☐<br>Racando ☐<br>Cro Cro ☐<br>Marlin ☐<br>Other (specify) ☐<br>________________	Flying fish ☐<br>Swordfish ☐<br>Moonshine ☐<br>Barracuda ☐<br>Catfish ☐<br>Wahoo ☐<br>Mahi-mahi ☐<br>Herring/cod ☐<br>Cutlass fish ☐<br>'Bechin' ☐<br>Sardines ☐<br>'Fried dry' ☐
Do you eat shark?	Yes ☐<br>No ☐	
Does anyone in your household eat shark meat?	Yes ☐<br>No ☐	
If shark is eaten How often do you/they eat shark?	Less than once a month ☐<br>Once a month ☐<br>Fortnightly ☐<br>Once a week ☐<br>Twice a week ☐<br>3 times per week ☐<br>Every day ☐	

	Other (specify) ☐ ______________	
If shark is eaten What generally influences your/their purchase of shark?	Cost ☐<br>Taste ☐<br>Look e.g.colour ☐<br>Odour ☐<br>Availability ☐<br>Custom/cultural ☐<br>**Amount of bones** ☐<br>Health benefits ☐<br>Type of dish ☐<br>Easy to prepared ☐<br>Media/advertising ☐<br>Suggestion by family/friend ☐<br>Other (specify) ☐<br>_________________	Question applicable to those who buy to eat at home **<u>and</u>** those who purchase from bake and shark vendors /restaurants.<br><br>**Amount of bones** = This is a major deciding factor when choosing fish in Tobago and Trinidad (personal observation).
If shark is eaten Where do you/they buy this shark?	Fishing depot ☐<br>Market ☐<br>Grocery ☐<br>Restaurant ☐<br>Food/street stand ☐<br>e.g. Maracas S&B sellers	Fish vendor ☐<br>Personal catch ☐<br>Frozen seafood retailers ☐
If shark is eaten Do you/they buy it to prepare at home?<br><br><br>*If shark is prepared at home* What form do you/they buy shark to prepare at home for eating?	Yes ☐<br>No ☐<br>Not sure ☐<br>________________________<br><br>Frozen uncooked (slices/fillets/whole) ☐<br>Fresh (fillet/slices/ whole) ☐<br>Precooked frozen ☐<br>Dried/dehydrated ☐<br>Salted ☐<br>Other (specify) ☐<br>_______________	
If shark is prepared at home How many pounds of shark meat, do you/they usually buy in a single purchase?	Less than 1 pound ☐<br>1 -1.5 pounds ☐<br>2 - 2.5 pounds ☐<br>3 - 4 pounds ☐<br>> 4 pounds ☐	Question applicable to those who buy shark **<u>to prepare at home</u>**. Categories based on minimum selling unit in local produce and famer markets (personal observation).

If shark is prepared at home How do you/they cook shark meat?	Curried ☐<br>Stewed ☐<br>Baked ☐<br>Grilled ☐<br>Steamed ☐<br>Boiled (e.g. in broths, soups) ☐<br>Fried (e.g. for 'bake and shark') ☐<br>Other (specify) ☐<br>____________________	Question applicable to those who buy shark **<u>to prepare at home</u>**.<br><br>Interviewee can select **<u>more than one</u> option.**
If shark is eaten How often do you/they buy shark?	Less than once a month ☐<br>Once a month ☐<br>Once fortnightly ☐<br>Once a week ☐<br>Twice a week ☐<br>3 to 4 times a week ☐<br>Every day ☐	Question applicable to those who buy to eat at home **<u>and</u>** those who purchase from bake and shark vendors /restaurants.
If you were made aware of heavy metals being present in the fish you bought, what would you do with the fish?		
If you were made aware that some commonly eaten shark species in Trinidad and Tobago were endangered, would you change your consumption habits/ tell others to change their shark consumption habits?<br><br>*If **Yes** to change* What would you switch to/ tell others to switch to?		

*If **No** to change* Can you tell me what your reason is for not switching/not telling others to change their consumption habits?
