# Supplementary material for: An evaluation of the public’s Knowledge, Attitudes and Practices (KAP) in Trinidad and Tobago regarding sharks and shark consumption
Source: PLoS One. 2020 Jun 9;15(6):e0234499. doi: 10.1371/journal.pone.0234499 (PMC7282724; doi:10.1371/journal.pone.0234499)
Supplement: S8 Appendix — (PDF) [file pone.0234499.s008.pdf]

Results of univariate and multivariate logistic regressions predicting knowledge concerning threats to sharks and the status of sharks in Trinidad and Tobago.

Demographics	N	Correct Response N (%)	COR (95% CI)	AOR (95% CI)
**Gender**				
Male	267	111 (41.6)	1	
Female	294	104 (35.4)	0.77 (0.55, 1.08)	
**Age Range**				
<20	21	6 (28.6)	1	
20-29	183	87 (47.5)	2.27 (0.84, 6.10)	
30-39	116	50 (43.1)	1.89 (0.69, 5.23)	
40-49	100	34 (34.0)	1.29 (0.46, 3.62)	
50-59	78	22 (28.2)	0.98 (0.34, 2.86)	
≥60	64	17 (26.6)	0.90 (0.30, 2.71)	
**Education**				
Primary or None	57	14 (24.6)	1	1
Secondary	201	71 (35.3)	1.68 (0.86, 3.27)	1.64 (0.83, 3.24)
Tertiary	300	126 (42.0)	2.22 (1.17, 4.24)*	2.31 (1.20, 4.46)*
**Employment**				
Employed	347	134 (38.6)	1	
Not Employed	209	76 (36.4)	0.91 (0.64, 1.30)	
**Island**				
Trinidad	473	162 (34.2)	1	
Tobago	94	55 (58.5)	2.71 (1.72, 4.26)*	2.81 (1.78, 4.46)*
**Area of Residence**				
Urban	326	124 (38.0)	1	
Rural	228	84 (36.8)	0.95 (0.67, 1.35)	

*Indicates that the associated demographic category was found to be a significant predictor of knowledge.
