# Supplementary material for: An evaluation of the public’s Knowledge, Attitudes and Practices (KAP) in Trinidad and Tobago regarding sharks and shark consumption
Source: PLoS One. 2020 Jun 9;15(6):e0234499. doi: 10.1371/journal.pone.0234499 (PMC7282724; doi:10.1371/journal.pone.0234499)
Supplement: S9 Appendix — (PDF) [file pone.0234499.s009.pdf]

Results of univariate and multivariate logistic regressions predicting attitudes concerning consumption of shark meat, consumption and fishing of endangered shark species, and labelling of shark meat, among residents of Trinidad and Tobago.

Demographics	N	Attitudes Supporting Reduced Impact N (%)	COR (95% CI)	AOR (95% CI)
**Gender**				
Male	267	187 (70)	1	1
Female	294	230 (78.2)	1.54 (1.05, 2.25)*	1.50 (1.02, 2.20)*
**Age Range**				
<20	21	14 (66.6)	1	
20-29	183	151 (82.5)	2.36 (0.88, 6.31)	
30-39	116	91 (78.4)	1.82 (0.66, 4.99)	
40-49	100	71 (71.0)	1.22 (0.45, 3.35)	
50-59	78	51 (65.4)	0.94 (0.34, 2.62)	
≥60	64	39 (60.9)	0.78 (2.28, 2.20)	
**Education**				
Primary or None	57	44 (77.2)	1	
Secondary	201	141 (70.1)	0.69 (0.35, 1.38)	
Tertiary	300	229 (76.3)	0.95 (0.49, 1.87)	
**Employment**				
Employed	347	253 (72.9)	1	
Not Employed	207	160 (76.6)	1.21 (0.82, 1.81)	
**Island**				
Trinidad	473	362 (76.5)	1	1
Tobago	94	60 (63.8)	0.54 (0.34, 0.87)*	0.56 (0.35, 0.90)*
**Area of Residence**				

Urban	326	241 (73.9)	1	
Rural	228	171 (75.0)	1.06 (0.72, 1.56)	

*Indicates that the associated demographic category was found to be a significant predictor of attitude.
