# Supplementary material for: An evaluation of the public’s Knowledge, Attitudes and Practices (KAP) in Trinidad and Tobago regarding sharks and shark consumption
Source: PLoS One. 2020 Jun 9;15(6):e0234499. doi: 10.1371/journal.pone.0234499 (PMC7282724; doi:10.1371/journal.pone.0234499)
Supplement: S10 Appendix — (PDF) [file pone.0234499.s010.pdf]

Results of univariate logistic regressions predicting practices concerning consumption of shark meat, consumption and fishing of endangered shark species, labelling of shark meat, and consumption of seafood containing heavy metals among residents of Trinidad and Tobago.

Demographics	N	Reduced Impact Practices N (%)	COR
**Gender**			
Male	267	111 (41.6)	1
Female	294	156 (53.1)	1.59 (1.14, 2.22)*
**Age Range**			
>20	21	14 (66.7)	1
20-29	183	75 (41.0)	0.35 (0.13, 0.90)
30-39	116	50 (50.0)	0.50 (0.19, 1.33)
40-49	100	52 (52.0)	0.54 (0.20, 1.46)
50-59	78	44 (56.4)	0.65 (0.24, 1.79)
≥60	64	26 (40.6)	0.34 (0.12, 0.96)
**Education**			
Primary or None	57	31 (54.4)	1
Secondary	201	94 (46.8)	0.74 (0.41, 1.33)
Tertiary	300	141 (47.0)	0.74 (0.42, 1.31)
**Employment**			
Employed	347	162 (46.7)	1
Not Employed	207	103 (49.8)	1.13 (0.80, 1.60)
**Island**			
Trinidad	473	222 (46.9)	1
Tobago	94	48 (51.1)	1.18 (0.76, 1.84)
**Area of Residence**			
Urban	326	159 (48.8)	1

Rural	228	107 (46.9)	0.93 (0.66, 1.30)

*Indicates that the associated demographic category was found to be a significant predictor of practices.
